# The effect of surface treatment and thermal aging on the bonding of clear aligner attachments to provisional resin-based material: shear bond strength analysis

**DOI:** 10.3389/froh.2024.1449833

**Published:** 2024-07-30

**Authors:** Suliman Y. Shahin, Norah AlQahtani, Tahani H. Abushowmi, Intisar A. Siddiqui, Sultan Akhtar, Essam A. Nassar, Mohammed M. Gad

**Affiliations:** ^1^ Department of Preventive Dental Sciences, College of Dentistry, Imam Abdulrahman Bin Faisal University, Dammam, Saudi Arabia; ^2^ College of Dentistry, Imam Abdulrahman Bin Faisal University, Dammam, Saudi Arabia; ^3^ Fellowship Program in Prosthodontics, College of Dentistry, Imam Abdulrahman Bin Faisal University, Dammam, Saudi Arabia; ^4^ Department of Dental Education, College of Dentistry, Imam Abdulrahman Bin Faisal University, Dammam, Saudi Arabia; ^5^ Department of Biophysics, Institute for Research and Medical Consultations (IRMC), Imam Abdulrahman Bin Faisal University, Dammam, Saudi Arabia

**Keywords:** clear aligner attachments, surface treatment, bond strength, provisional prostheses, composite

## Abstract

**Objectives:**

The aim of this study is to evaluate the effect of different surface treatments on the shear bond strength (SBS) of clear aligner attachments bonded to Bis-acryl provisional crowns.

**Methods:**

120 cylindrical bisacrylic composite material (ProTemp type) specimens were prepared and divided into six groups (*n* = 20) based on surface treatment, control: (no treatment); super coarse grit diamond bur, carbide bur, alumina-blasting, non-thermal plasma treatment, and Er:YAG laser treatment. The features of treated surfaces were examined using scanning electron microscopy (SEM). A flowable composite resin (Transbond XT; 3M Unitek) was bonded to the specimens forming the attachment. Half of specimens were subjected to thermal cycling (5,000 cycles). SBS was measured before and after thermal cycling. Each specimen was loaded at the attachment/resin interface at a speed of 0.5 mm/min until failure. The nature of the failure was analyzed using the composite remnants index (CRI). Two-way ANOVA and Tukey HSD were used for data analysis *α *=  0.5. For CRI scores analysis, Kruskal-Wallis test and Dunn's multiple comparison were used as *post-hoc* test.

**Results:**

SEM analysis showed that all surface treatments altered surface properties and increase surface bonding area. The specimens treated with plasma, Er:YAG laser, and alumina-blasting had higher SBS values before and after thermal cycling. In comparison to control plasma, Er:YAG laser, and alumina-blasting showed a significant increase in SBS (*P* < 0.001) while carbide and diamond bur groups showed no significant differences (*P* > 0.05). Thermal cycling significantly decreased the SBS of control, carbide bur, diamond bur, and Er:YAG laser while no significant effect of alumina-blasting and plasma group. Er:YAG laser and plasma groups significantly exhibited more dominance for scores 2 and score 3 and the absence of score 0.

**Conclusion:**

Alumina-blasting, Er:YAG laser, or non-thermal plasma surface treatments increased the shear bond strength between clear aligner attachments and resin-based restorations.

## 1 Introduction

There is an increase in the number of patients seeking orthodontic treatment, many of whom require an interdisciplinary approach. In turn, this led orthodontists to seek out various treatment modalities (1). In some orthodontic cases, collaboration with other specialties during treatment procedures is required for proper treatment outcomes (2). Among these dental procedures is provisional restorations, which is used when some teeth require movement but the crown cannot be used due to tooth fracture or extensive carious lesions that have spread subgingivally. In these cases, a provisional restoration is required to temporarily restore the tooth before orthodontic root extrusion in order to mitigate any biological width violation that might occur in final crowns (3). Provisional crowns are made of various resins, including Polymethylemethacrylate (PMMA) resin and Bis-acryl composite resin. Because of its advantages over PMMA, Bis-acryl composite resin is the most commonly used for provisional crown fabrication (4, 5). The benefits are numerous including: low exothermic reaction while setting, good strength, good marginal adaptation and stability of the color of the crowns (6).

The amount of force required for clinical tooth movement is an important consideration in orthodontic treatment. The amount of force required for tooth movement varied upon the type of tooth movement (15–120 g) (3, 7). The bond strength between brackets and tooth structure/restorations is a determining factor in orthodontic treatment success and completion within the time frame specified (2, 3). According to the literature, the minimum bond strength required for orthodontic tooth movement is between 6 and 8 MPa (3, 7).

Removable aligners are becoming more popular as technology advances, owing primarily to their aesthetic appeal (8, 9). Clear aligners are thermoplastic removable appliances worn by patients in sequence to achieve the desired outcome (10). The main disadvantage of the removable clear aligner was that it was difficult to control tooth movement in some cases, such as extrusion, rotation, or root movement control (11). As a result, the “attachment” composite button was introduced. These attachments are bonded to tooth surfaces and aid in controlling tooth movement when using a removable clear aligner (10, 12, 13).

Due to the importance of composite attachments in orthodontic treatment when using removable clear aligners, ideal attachment material characteristics such as ease of application, high wear resistance, and satisfactory bond strength have been reported (9). Several studies have been conducted to select attachment material and bonding features for various tooth surfaces as well as different restorative materials (9, 14). Surface treatment has a direct impact on bonding in clear aligner therapy, affecting treatment outcomes and necessitating a dental visit for a new or lost attachment (9, 15). A weak bond between attachments and resin-based resin increases the bonding failure rate and may negatively affect the treatment progress, as well as increase the cost and patient discomfort (2, 15).

Several methods have been proposed to improve bonding strength via increased surface area at resin interfaces, including chemical, mechanical, and combination approaches (6, 16). In addition to surface treatment, specimens aging affect the bond strength, as reported by Chay et al. (6) Air abrasion with aluminum oxide particles, bur roughening, and Er:YAG laser are among the surface treatments available. For temporary crown roughening, laser and plasma, acid etching, hydrofluoric acid etching, or combinations were proposed (1, 16–18). The Er:YAG (erbium-doped: yttrium aluminum garnet) laser has been used for a variety of purposes, including surface treatment because of its ability to roughen resin-based materials and improve bonding by increasing micromechanical interlocking (15, 17, 18). Meanwhile, non-thermal plasma exhibited acceptable ranges of 7–14 MPa when applied to enamel surface before bonding (18). The mechanical properties of composite resin can be influenced by hydrolytic degradation (19). In *in vitro* studies, deem the long-term water storage and thermal cycling as pertinent conditions for testing the durability of resin bonds (20).

Previous studies using different surface treatments have not investigated the bond strength of clear aligner attachments to rein-based materials. As a result, the current study aimed to assess the influence of various surface treatment (Alumina-blasted, bur grinding, Er:YAG laser, and non-thermal plasma) and the thermal cycling on the shear bond strength (SBS) between clear aligner attachment and a resin-based). The null hypothesis was stated as “There is no effect of the resin surface treatment modality and thermal cycling on SBS between clear aligner attachments- using Invisalign templates- and resin-based restorations.”

## 2 Materials and methods

Sample size calculation was done by calculating the difference in mean SBS for the groups using Fuji Ortho LC brackets in MPa (9.33, 7.42), SD = 1.73 as reported by Rambhia et al. (3) on a significance level of *α* = 0.05 and power = 0.80% and 95% confidence level, the sample size per group needed was 20 per group (10 without thermal cycling and 10 after thermal cycling).

According to the manufacturer's instructions, 120 cylindrical specimens of bisacrylic composite material (ProTemp type) (cold curing temporary crown material; success CD, Neumünster/Germany) with 10 mm diameter and 15 mm height were prepared. Bisacrylic material from automix cartridges was injected into a standardized silicon mold (10 mm × 15 mm), and the excess material was extruded by pressing it with a glass slab. For each specimen curing, the tip of the optical guide of the light cure was directly positioned on the specimen's surface for 30 s. (Acteon Satelec mini LED light cure) at 1,250 mW/cm^2^ rapid mode intensity. Following that, all specimens were stored in distilled water at 37 °C for 24 h.

The specimens were grouped into six groupings (*n* = 20) according to surface treatment (Table 1). (Control group) no surface treatment (C). Group I, super coarse grit diamond bur (DB). Group II, carbide bur (CB). Group III alumina-blasting (50-μm alumina particles) by applying 0.55 MPa of propulsion pressure (Wassermann Dental-machine, CEMAT-NT3, GMBH, Hamburg, Germany) for 10 s from a distance of 10 mm. (SB, *n* = 20). Group IV, Non-thermal plasma treatment (NTPT. Group V, Er,Cr:YSGG laser (Waterlase Express; Biolase, Irvine, USA) (L. For standardization of the treatment process and direction of the bonded treatment area, a plastic cover was customized on the prepared disc surface. The plastic cover has an exposed area of 5 × 2 mm at the center and was positioned per specimen during treatment.

**Table 1:** Material grouping according to surface treatment and surface treatment protocols.

Group (*n* = 20)	Surface treatment
Control	No treatment.
Black, supercoarse grinding	One skilled investigator used a diamond bur (Jota diamond bur, Switzerland) with one grit size: supercoarse and subjected each specimen to five strokes in the same direction at the center of the surface.
Carbide	Carbide bur was used for applying five strokes for each specimen in the same direction at the center of the surface.
Sandblasting	Specimens were sandblasted (50 mg alumina particles) for 10 s at a distance of 10 mm using a Wassermann Dental-Machine, CEMAT-NT3, GMBH, Hamburg, Germany, with a propulsion pressure of 0.55 MPa. Specimens were rinsed for 30 s with a constant stream of water before being dried with compressed air.
Non-thermal plasma treatment	Piezobrush (PZ4) relyon was used, Piezobrush (PZ4) is a compact plasma handheld device intended for use in laboratories, pre-development and assembly of small series. The Piezoelectric Direct Discharge (PDD®) technology is used to generate cold active plasma at a temperature below 50 °C. In order to increase the surface energy with high efficiency, and reduce germs and odors Plasma is used.
	Module standard was used distance: 5 mm, Time: 30 s, Power: 80% from the manual.
Er,Cr:YSGG laser (Waterlase Express; Biolase, Irvine, USA)	Tip used: S75/750 μm tips/Tip diameter: 0.6 mm (6 mm long) Wavelength: 2,780 nm power: 2.75 W, energy: 185–190 mJ frequency: 25 Hz (H) short pulse (60 μs air/fluid cooling: 60% air and 40% water time of irradiation: 30 s Distance: 5 mm

After complete surface treatment for all specimens, scanning electron microscopy (SEM) (TESCAN, VEGA3, Czech Republic) was used to assess the surface treatment effect. The treated specimens were gold sputter coated (Quorum, Q150 R ES) and then scanned under SEM at 20 kV with a working distance of ∼10 mm. Electronic images with different magnifications were recorded of treated specimens for surface analysis.

**Figure 1:**
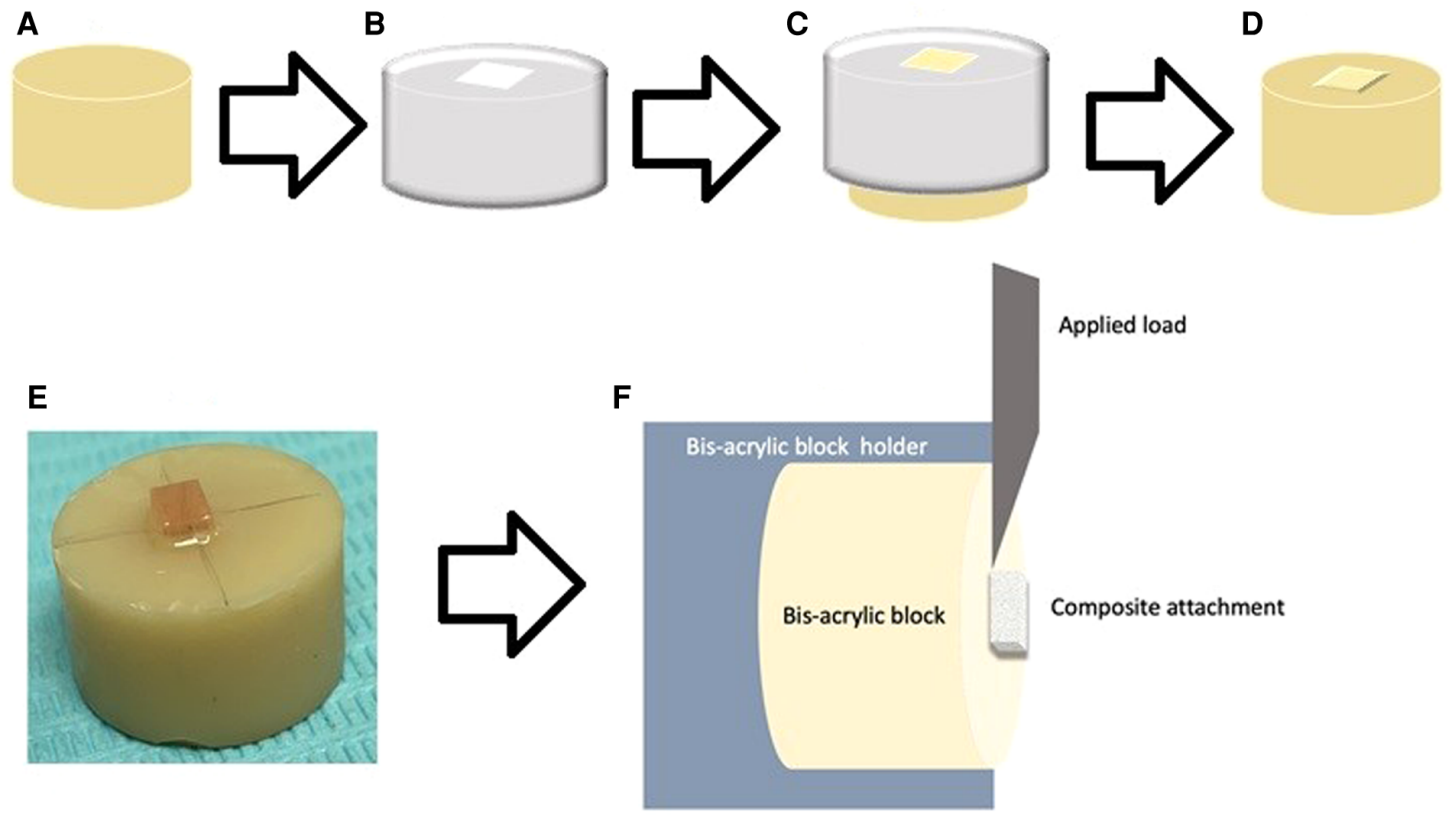
Illustrated diagram for specimens’ preparations and testing. (**A**) Cylindrical specimens of (10 mm × 15 mm) bisacrylic composite material (ProTemp type), (**B**) the Invisalign® template aligner with 2 × 3 mm rectangular attachment and composite bonding, (**C**) composite applied using the template (**D**, **E**) bonded flowable light-cured composite resin, (**F**) specimen loaded on the instron machine for SBS.

A flowable light-cured composite resin (Transbond XT; 3M Unitek) was bonded to the provisional material specimens (Figure 1A) to form the attachment. The attachment template used was unified for all attachments by using an Invisalign attachment template of a maxillary central incisor with a rectangular attachment sized 3 × 2 mm (Figure 1B) and was used to bond all the attachments (14). The attachment template was used to apply flowable composite followed by excess composite was removed using an explorer (Figure 1C). To ensure that the thickness of the attachments is uniform, a constant 5 N force was applied. The attachment was then light-cured for 10 s on both sides (5 s on each side) with an Ortholux XT Visible light-curing unit (3M Unitek), per the manufacturer's instructions (Figures 1D,E).

After bonding, the samples were stored in distilled water at 37 °C for 24 h. Half of specimens were incubated for 30 s in cold or hot water with a 5-s interval between successive immersions, using a thermocycling machine (Thermocycler THE-1100-SD Mechatronik GmbH, Feldkirchen—Westerham, Germany), applying 5,000 cycles to alternate 5 °C and 55 °C water baths.

**Figure 2:**
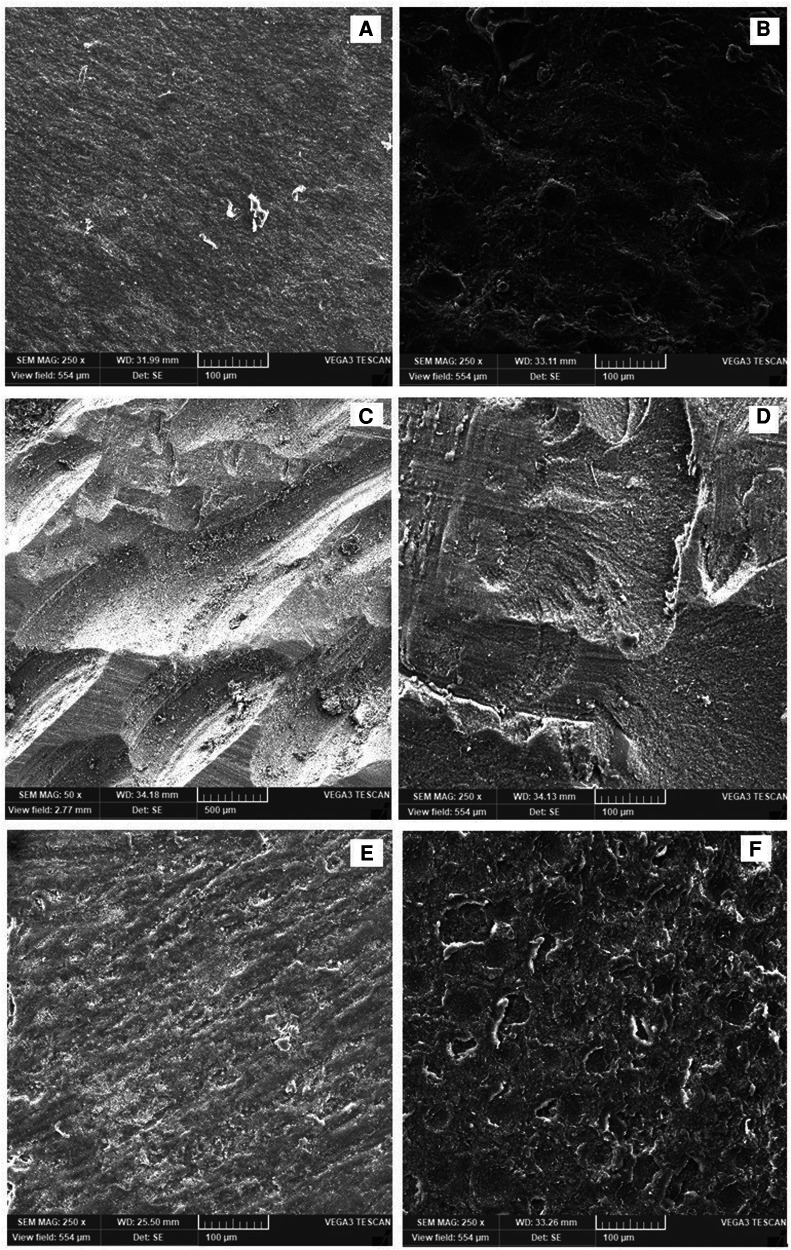
(**A**–**F**) Surface morphology examined by the SEM analyzing the effect of surface treatment: (**A**) control, (**B**) alumina-blasting, (**C**) carbide bur roughening, (**D**) diamond bur roughening, (**E**) plasma, (**F**) Er:YAG laser.

For SBS measurements, each specimen was fixed in the customized jig on the testing machine (Instron 8871; Instron Co., Norwood, MA). The load was applied using a beveled blade at the composite attachment/resin interface at a speed of 0.5 mm/min until failure (Figure 2F). The failure load (N) was used to count SBS (MPa) using this equation: SBS = F/A, where F is the debonding force in Newton, and A is the cross-sectional surface area of the attachment base in square millimeters (15).

An optical microscope (Nikon, H550L, Tokyo, Japan) at 10-fold magnification was used to assess the surface of the debonded provisional material and the debonded composite attachment. The composite remnants index (CRI) score on the crown surface was used to decide the nature of failure which was classified as described in previous studies (2, 16, 21, 22): 0 = no composite left on the crown, 1 = lower than 50% of composite left on the crown, 2 = more than 50% of composite left on the crown, 3 = 100% of composite left on the substrate resin.

### 2.1 Statistical analysis

Data analyses were carried out by using SPSS-25.0 (IBM product, Chicago, USA). Shapiro test of normality was performed to assess the distribution of data to apply the appropriate test. It revealed that the sample was found to be normally distributed. Mean and standard deviation were calculated for presentation of shear bond strength. Two-way ANOVA was performed to compare the results of shear bond strength between the groups, followed by Tukey HSD for pairwise comparison. For comparison of ordinal data analysis based on CRI scores, Kruskal-Wallis test was used to compare between groups (surface treatment), and Duncan's multiple range test was used as *post-hoc* test. A *p*-value ≤0.05 was considered statistically significant.

## 3 Results

As shown in Figure 2, all surface treatments result in specimens' surface roughness. However, each treatment process produced different kinds of surface morphological features. For example, alumina-blasting method resulted in several irregularities with some uniform pits represent abrasive particles (Figure 2B). Bur treatment resulted in serration and oriented groove with carbide bur (Figure 2C), while faint irregular groove resulted when using a diamond bur (Figure 2D). Plasma treatment showed the lowest roughens between all surface treatments (Figure 2E). The Er:YAG laser treated specimens exhibited surface irregularities that was comparable with the alumina-blasting group in which more irregularities and distributed small pits were found (Figure 2F).

The mean and standard deviations of SBS between the tested groups before and after thermal cycling are presented in Table 2. The specimens treated with plasma, Er:YAG laser, and alumina-blasting showed higher SBS values (10.69 ± 3.56, 9.68 ± 2.03, 9.15 ± 3.29 MPa respectively).

**Table 2:** Mean and SD of shear bond strengths between tested groups declaring the treatment's effects and thermal cycling.

Thermal cycling	Surface treatment						*P* value
	No treatment	Alumina-blasting	Carbide bur	Diamond bur	Plasma	Er:YAG laser	
NTC	7.32 ± 1.03^a^	10.93 ± 2.56^b^	7.85 ± 2.1^a^	7.43 ± 3.3^a^	10.99 ± 3.1^b^	11.02 ± 4.2^b^	0.001
TC	5.98 ± 0.90^a^	9.15 ± 3.29^b^	5.57 ± 3.07^a^	5.61 ± 4.43^a^	9.69 ± 3.56^b^	8.68 ± 2.03^b^	0.004
*P* value	0.007*	0.08	0.002*	0.005*	0.63	0.042*	

TC, thermocycling; NTC, no thermocycling.

* Significant difference *p *< 0.05.

^a,b^ The same small letters per raw indicated insignificant pairwise between groups *P* > 0.05.

In comparison to the control group; plasma, Er:YAG laser, and alumina-blasting treatment showed a significant increase in SBS (*p* < 0.05). While carbide and diamond bur groups showed no significance differences with the control group (*P > *0.05).

Among surface treatment group, plasma, Er:YAG laser, and alumina-blasting significantly showed significant increase in SBS compared with the carbide and diamond bur groups (*p* < 0.05). Results showed no significance difference between carbide bur and diamond bur group which showed the lowest SBS values between surface treatment groups (7.85 ± 2.1 MPa and 7.43 ± 3.3 MPa) before thermal cycling and (5.57 ± 3.07 MPa and 5.61 ± 4.43 MPa) after thermal cycling.

In regards to thermal cycling showed a significant decrease in SBS in all groups except alumina-blasted group (*p* = 0.08) and plasma group (*P* = 0.63). Moreover, no treatment, carbide bur, and diamond bur groups showed the lowest SBS values after thermal cycling.

**Figure 3:**
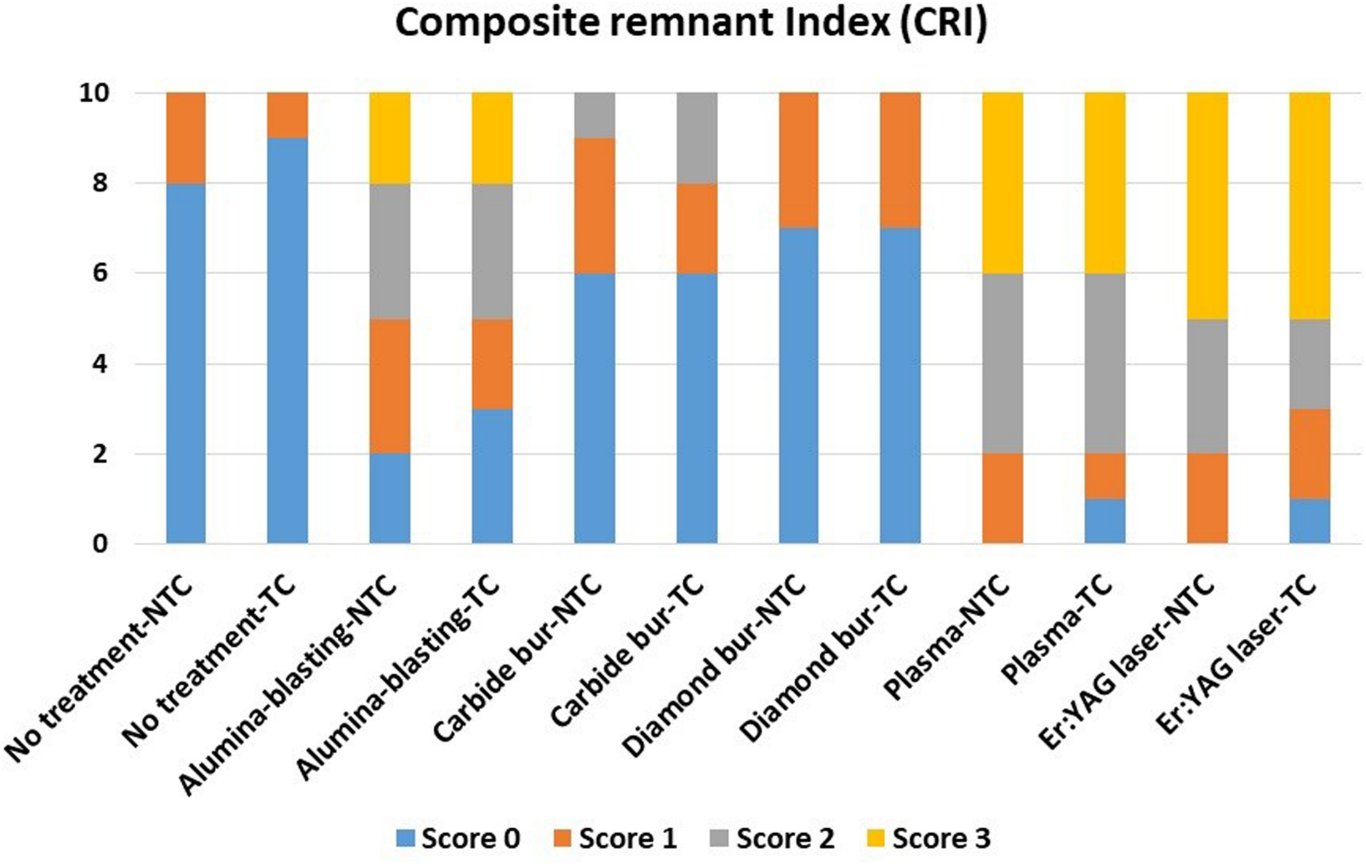
The composite remnant index (CRI) scores and significance between all tested groups. Score 0 = no composite left on the specimen surface. Score 1 = less than half of the composite left. Score 2 = more than half of the composite left. Score 3 = all bonded area of the surface of the specimen covered with composite.

CRI scores are presented in Figure 3. A statistical test (Kruskal-Wallis test with Duncan's multiple range *post-hoc* test) was performed to compare the different CRI scores. The effect of thermal cycling per score for each surface treatment showed no significance different. Comparing scores between different groups, Score 0 is significantly increased with no treatment, carbide bur, and diamond bur groups while score 2 and 3 were significantly increased with plasma, Er:YAG laser group without significant between plasma vs. Er:YAG laser groups. Alumina-blasting group showed no significant differences with equal distributions between CRI scores.

## 4 Discussion

It is critical to have sufficient bonding between the composite attachment and the temporary restorations which will allow the treatment to be completed on time and with accurate results (1). Debonding and failure between attachment and crown necessitate additional visits and costs, and may jeopardize treatment success (6, 8). There have been a few studies done with clear aligners to improve the bond strength between attachments and resin-based temporary crowns. As a result, this study proposed various surface treatments (alumina-blasting, bur roughening, Er:YAG laser, and plasma) as well as the thermo-cycling effect. The findings if this study demonstrated that alumina-blasting, plasma, and Er:YAG laser increased SBS of attachment to resin-based restoration while burs roughening had no effect on the SBS.

To simulate clinical conditions and assessing the behavior of bonded composite attachment with different surface treatment, all specimens with bonded attachments were subjected to thermal stress. Thermal cycling is considered to be a guideline for material behavior in the oral cavity. Previous studies subjected specimens to thermal cycling with variety of cycles and temperatures (2, 21, 23). In present study bonded specimens were subjected to 5,000 cycles as repowered in previous studies (16, 23) simulating 6 month clinical usage. Our findings showed that thermal cycling significantly decrease the SBS in all groups except alumina-blasted and plasma groups. Moreover, in control, carbide bur, and diamond bur groups showed the lowest SBS values after thermal cycling. This aging procedure affects bond strength, particularly at the resin-based restoration/attachment interface (24). Swelling caused by water sorption and thermal stress (due to difference in the coefficient of thermal expansion of materials) as well as hydrolytic degradation of hydro­philic elements in adhesives have a direct negative impact on shear bond strength (21, 23, 24). Moreover, increased temperature has been shown to accelerate water uptake (25).

To the best of the authors' knowledge, no study has been conducted to assess the effect of Er:YAG laser and plasma treatments on the roughening of resin-based restorations used as provisional crowns. According to SEM findings, Er:YAG laser applications resulted in a rougher surface with microporosities and microretention areas, which increased the bonding surface area (17, 26). Although plasma treatment produces a rough surface, it is not the same as Er:YAG laser treatment. Furthermore, plasma application resulted in surface washing, degreasing, and activation (20, 27). Plasma activated the chemical bonds on the treated surface, resulting in the formation of oxygen-containing functional groups (C = O and -OH), which resulted in a hydrophilic surface and, ultimately, increased surface wettability (27). Increased wettability resulted in improved martial flow and uniformity across the resin surface (28).

Different surface treatments suggested in the literature, such as bur roughening and air-abrasive particles, were included in the current study to confirm plasma and Er:YAG laser applications (15, 16). Er:YAG laser and plasma treatments showed adequate shear bond strength. When the surface treatment effects were compared, the Er:YAG laser produced a rougher surface. However, both treatments demonstrated high shear bond strength. This could be due to the plasma's ability to affect the wettability of specimen surfaces, which is consistent with previous research (26). This provided a good opportunity for composite material to bond easily, resulting in satisfactory shear bond strength (26). Yildirim compared the bond strength between soft liner and denture base after plasma and Er:YAG laser treatments and found that both significantly increased bond strength. Zarif Najafi et al. (29) demonstrated that bond strength is surface treatment type dependent, and recommended Er:YAG laser irradiation to improve the bond strength between brackets and provisional crown in a previous study (29). Goymen et al. (30) discovered an increase in shear bond strength of brackets to protemp temporary crowns after Er:YAG laser irradiations, but the value was very low (5.43 MPa) when compared to the current study. This could be because of the material differences in composite attachment vs. orthodontic brackets. Orthodontic brackets add a metal-composite interface to the composite-resin interface. Whereas, in clear aligner attachments, there is a unified composite-resin interface, likely, increasing the shear bond strength of the specimens.

The shear bond strength between flowable composite attachments and resin-based restoration was significantly increased by the alumina-blasting treatment. This was due to the treatment with air-abrasive particles, which resulted in a more irregular surface rather than a smooth surface (16). These irregularities increased the surface bond area while also forming grooves and pits for micromechanical locking of composite and crown materials (29). Furthermore, this finding is consistent with previous studies (2, 16) that found that alumina—blasting with alumina particles increased shear bond strength between composite and resin-based restorations, as well as between temporary crowns and metallic brackets (2, 22, 29).

Previous research has suggested that bur roughening improves shear bond strength (1, 2, 16, 23), while other studies found no difference (31, 32). In the current study, bur roughening produced a rough surface, but shear bond strength remained unchanged and was comparable to the untreated group. Although diamond and carbide burs roughening had an effect, no changes were observed due to the faint grooves created by bur roughening, which resulted in serrations and macro-retentive areas (23) rather than micromechanical retention when compared to other surface treatments (32) Furthermore, the method and frequency of bur application could be another explanation, necessitating more invasive grinding procedures (32).The present study's findings on bur roughening contradict previous studies (1, 2, 16) which found that bur roughening increased the shear bond strength between provisional crown and metal brackets when compared to untreated. The differences in results could be attributed to differences in methodology, such as the type of bur used for roughening, the direction and frequency of bur strokes and the composite type, as well as the use of metal brackets instead of the composite attachment used in the current study.

For provisional crown fabrication, the two major categories are methacrylate resins and composite-based materials (24). Protemp [bisphenol A glycerolate dimethacrylate (Bis- GMA)] is a resin-based restorative that has been studied as a temporary and its bonding with different resins (16, 33) There is no information in the literature about the shear bond strength of clear aligner attachment to Bis-GMA. Rambhia et al. (3) and Goymen et al. (30) assessed the bond strength of brackets to various temporary crown materials, finding that the shear bond strength of brackets ranged from 2.81 to 9.65 MPa and the SBS of Protemp ranged from 8.33 to 9.65 MPa. Another study reported a low shear bond strength of protemp (3.68 MPa) (30) despite the fact that the bonded specimens were kept in a thermocycling machine for 500 cycles, which could explain the low SBS.

Flowable composites (FC) are a type of dental resin with low viscosity and the capacity to be applied in narrow spaces. D’Antò et al. (34) have shown that various types of dental composite viscosities do not affect the attachment shape of extracted teeth when using aligner templates. Lin Et al. (35) clinically compared the survival rate between a flowable composite and a packable composite for Invisalign aligner attachment and concluded was no significant difference between the two composites and using flowable composite may save time. The mechanical properties of low-viscosity flowable composites are similar to packable composites, but the injector design of flowable composites is more suitable for clinical use. Also, reports have shown that flowable composites have higher bond strength than packable composites (36) and surpassed the clinically accepted value of 6–8 MPa (37). Furthermore, flowable composites require less time for application than high viscosity composites (37). Flowable composite was chosen for this study because it is a commonly used method for placing clear aligner attachment using Invisalign. This is particularly important to ensure that the composite follows the attachment template shape and enters the roughened surface, resulting in good mechanical properties. Clinically, attachments are subjected to wear force during insertion and removal, which should be considered in future studies (different attachment composite materials and new resin-based restoration fabricated with digital technology; CAD-CAM milled and 3D printed).

In line with previous researches (1, 31) CRI score 0 was found frequently in control and burs groups with low shear bond strength values. While scores 1 and 2 varied for air-abrasive, plasma, and Er:YAG laser groups, score 3 was dominant for Plasma and Er:YAG laser groups. The higher the CRI score, the more adhesive remnant on the crown surface bonding area (17). CRI results confirm the current study's findings, which are consistent with Dehghani et al. (17). This discovery confirmed the link between CRI score and shear bond strength. Considering the clinically acceptable shear bond strength required (6.5–10 MPa) (3, 7), bonding for orthodontic tooth movement and resistance to intraoral conditions is required. Air-abrasion, Er:YAG laser, and plasma applications demonstrated high shear bond strength greater than 9 MPa in the current study. For clear aligner attachments, clinically, surface treatment of resin-based restorations with alumina-blasting, Er:YAG laser, or plasma treatment can be recommended.

Although the specimens were aged and thermally cycled, the absence of oral conditions such as saliva, enzymes, beverages, dietary intake, and the generated force on the attachment was considered limitation of the current study. Another limitation is the use of only one resin for temporary restorations and one composite attachment material. As a result, future research on different brands of temporary resins and different fabrication methods (CAD-CAM provisional) with different surface treatments and different attachment composite resins in simulated oral conditions is recommended for future investigations. Recommendations for practice include the use of alumina blasting, Er:YAG laser, or non-thermal plasma treatments to enhance the shear bond strength of clear aligner attachments to resin-based restorations, especially in cases where long-term use of provisional crowns is needed, such as complex and lengthy orthodontic treatment.

## 5 Conclusions

All surface treatments alter surface properties and increase the surface bonding area. However, shear bond strength of aligner composite attachments was significantly increased by surface treatment of resin-based restoration with Alumina-blasting, Er:YAG laser, or non-thermal plasma. Shear bond strength did not change after diamond and carbide burs roughening. However, thermal cycling has adverse effect on control, bur treatment, and laser treatment.

## Data Availability

The original contributions presented in the study are included in the article/Supplementary Material, further inquiries can be directed to the corresponding author.
